# Possible Association of Etanercept, Venous Thrombosis, and Induction of Antiphospholipid Syndrome

**DOI:** 10.1155/2014/801072

**Published:** 2014-05-15

**Authors:** Shanti Virupannavar, Anthony Brandau, Carla Guggenheim, Heather Laird-Fick

**Affiliations:** ^1^Internal Medicine, Michigan State University, 138 Service Road, A225 Clinical Center, East Lansing, MI 48825, USA; ^2^Internal Medicine and Hematology/Oncology, McLaren of Greater Lansing, 401 West Greenlawn Avenue, Lansing, MI 48910, USA; ^3^Internal Medicine and Rheumatology, Michigan State University, 138 Service Road, A225 Clinical Center, East Lansing, MI 48825, USA

## Abstract

Tumor necrosis factor **α** (TNF **α**) inhibitors are commonly used for treatment of aggressive rheumatoid arthritis and other rheumatic diseases. Etanercept is one of the medications approved for treatment of rheumatoid arthritis. Though many studies have documented the safety and efficacy of these medications, evidence for adverse effects is emerging including cancer, infections, and cardiovascular disease. There have been studies showing that these medications induce autoantibody production, including antinuclear antibodies and anti-dsDNA antibodies. Limited data exists, however, regarding induction of antiphospholipid antibodies (APLs) by TNF **α** inhibitors, including anticardiolipin antibodies (ACLs), lupus anticoagulant (LAC), and anti-**β**_2_-glycoprotein I (anti-**β**_2_ GPI), or an association between antibody development and clinical manifestations. In this case series, we describe five patients who developed venous thromboembolism (VTE) and APLs while receiving etanercept therapy. All five of our patients met the criteria for diagnosis of APS after receiving etanercept. Our case series supports the association between etanercept, APLs, and VTE. We believe that testing for APLs prior to initiation of anti-TNF therapy is reasonable, given this relationship and the risks associated with VTE.

## 1. Introduction

Tumor necrosis factor *α* (TNF *α*) is a cytokine essential to T-cell-mediated inflammatory responses [1–3]. TNF *α* inhibitors are now widely used for treatment of aggressive rheumatoid arthritis (RA) and other rheumatic diseases. Etanercept is one of the medications approved by the US Food and Drug Administration (FDA) for treatment of RA, psoriatic arthritis, and ankylosing spondylitis. It is a fusion protein produced by recombinant DNA which combines the ligand binding portion of human TNF receptor p75 with the Fc fragment of human IgG1, which binds soluble TNF *α*, working to block the action on its receptors [4, 5].m

While many studies have documented the safety and efficacy of these medications [1, 6, 7], evidence for adverse effects is emerging. These include infections, cancer, lymphoma, demyelinating disorders, and cardiovascular disease [1, 4, 8]. In addition, an association with these medications to autoimmune diseases has been reported [1, 4, 9–12].

TNF *α* agents induce autoantibody production, including antinuclear antibodies (ANAs) and anti-dsDNA antibodies [2, 4, 5, 11, 13–19]. Eriksson et al. [20] showed that ANA positivity increased from 24% of patients at baseline, to 77% at 30 weeks, and 69% at 54 weeks following initiation of a TNF *α* inhibitor. Many other studies have confirmed these findings. However, there are limited data regarding the induction of antiphospholipid antibodies (APLs) by TNF *α* inhibitors, including anticardiolipin antibodies (ACLs), lupus anticoagulant (LAC), and anti-**β**_2_-glycoprotein I (anti-**β**_2_ GPI). Two studies have reported antibody development in RA patients receiving etanercept. Jonsdottir et al. showed that up to 25% of such patients develop IgG or IgM ACL, while Ferraro-Peyret et al. showed that 21% developed APL positivity [17, 21]. The association between antibody development and clinical manifestations remains unclear [21–25].

In this case series, we describe five patients who developed venous thromboembolism (VTE) and APLs while receiving etanercept therapy.

## 2. Case Presentations

*Case  1*. A 67-year-old Hispanic man, with a nine-year history of RA, had been treated with multiple combinations of disease-modifying antirheumatic drugs (DMARDs) and corticosteroids. He had been receiving etanercept (25 mg twice weekly) for three years when he presented with dyspnea and right calf swelling. Duplex ultrasound confirmed right femoral deep venous thrombosis (DVT). Computed tomography angiography (CTA) of the chest showed massive filling defects in the right and left pulmonary arteries. He denied any personal or family history of hypercoagulable disorders, immobilization, smoking, trauma, blood clots, or heparin therapy. He had undergone an uncomplicated ankle surgery nine months prior to presentation. He had an elevated activated partial thromboplastin time (aPTT) of 48 seconds prior to initiating anticoagulation therapy. He tested positive for LAC and ANA, with speckled pattern at titer of 1 : 1280. ACL IgG and IgM were within normal range. LAC, ANA, and aPTT remained persistently elevated one year later. Further thrombophilia workup was negative.

*Case  2*. A 55-year-old Caucasian female with refractory RA had been on multiple DMARDs, including methotrexate, etanercept, rituximab, leflunomide, adalimumab, azathioprine, and minocycline. One year after restarting etanercept (50 mg every week), she presented to the emergency department with severe palpitations. She was found to have large bilateral VTE secondary to left lower extremity DVT. She had no risk factors for thrombosis. She had a positive ACL IgM, but LAC and the rest of the thrombophilia workup were negative. ACL IgM remained positive 12 weeks later. 

*Case  3*. An 85-year-old Caucasian woman with a remote history of tobacco use had been treated with methotrexate and etanercept for RA for three years when she presented with VTE in the absence of risk factors. She had a positive ACL IgG and LAC at presentation and 12 weeks later. Her aPTT was persistently elevated at 40.9 seconds. The other thrombophilia workup was negative. 

*Case  4*. A 53-year-old Caucasian female with seronegative inflammatory arthritis, poorly controlled by methotrexate, was started on etanercept add-on therapy. She had a negative workup for antiphospholipid syndrome (APS) prior to initiating the new medication. One year later she developed dyspnea and bilateral lower extremity swelling. She was found to have a left lower lobe VTE, along with left tibial DVT. Her only risk factor for thrombosis was a family history of DVT in her mother. At presentation she had a prolonged aPTT of 44 seconds and was positive for LAC. These labs remained persistently positive for more than three months. The other thrombophilia workup was negative. 

*Case  5*. A 61-year-old Caucasian male with psoriatic arthritis had been treated with efalizumab for two years for psoriasis skin involvement. Due to recurrent flares of his arthritis, efalizumab was discontinued. He was started on methotrexate 5 mg per week and prednisone 10 mg daily. After a 25-day washout period, etanercept was started. His arthritis symptoms improved after one week. Eventually methotrexate and prednisone were discontinued. Three years later, he presented with multiple DVTs. He had no risk factors for thrombosis. He had a prolonged aPTT at 51 seconds and a positive LAC and anti-**β**_2_ GPI. All three of these labs remained positive for more than three months. 

## 3. Discussion

Antiphospholipid syndrome is characterized by the occurrence of vascular thrombosis or pregnancy morbidity in the presence of APLs, which include ACL, anti-**β**_2_ GPI, and LAC [26]. According to the revised Sapporo criteria, APS can be diagnosed by one clinical criterion and at least one laboratory criterion. In order to meet the laboratory criteria, the APLs must remain positive on two or more occasions, at least 12 weeks apart [26]. All five of our patients met the criteria for diagnosis of APS after receiving etanercept. Prior to starting anticoagulation for VTE, all of them had tested for aPTT, APL, protein C, protein S, antithrombin III, Factor V Leiden, homocysteine, and prothrombin gene mutation. All had a prolonged aPTT and tested positive for at least one of the APLs prior to initiation of anticoagulation. When present, these tests remained positive for more than three months.

There are several proposed mechanisms by which TNF *α* inhibitors induce APL. One hypothesis is that the binding of an inhibitor to transmembrane TNF *α* induces apoptosis, leading to release of nuclear antigens and development of autoantibodies [5, 27]. Only infliximab binds transmembrane TNF *α*, however, so this hypothesis would not explain the autoantibodies seen with etanercept, which primarily binds soluble TNF *α* [5]. A second hypothesis is that TNF *α* inhibitors suppress the T-helper type 1 response, resulting in a T-helper type 2 response; this leads to autoantibody production and lupus-like features [12, 27]. Thirdly, TNF *α* inhibitors increase the likelihood for bacterial infections, which activate polyclonal B-lymphocytes and result in antibody production [12, 27]. Lastly, Masson suggests that TNF *α* inhibition results in overproduction of interferon *α*. This phenomenon, combined with individuals' predisposition to lupus-like syndromes and APS, leads to thromboembolic events [23].

Previous studies have not conclusively shown that TNF *α* blocker-induced APLs are associated with thrombotic events typical of APS [17, 21, 22, 25]. Two studies have found no association. Davies et al. [28] examined data from the British Society for Rheumatology Biologics Register and found that venous thromboembolism was not increased in patients receiving anti-TNF *α* treatment. Ferraro-Peyret et al. found no correlation between induction of autoantibodies (ACL or anti-**β**_2_ GPI) and development of APS or lupus-like syndrome over a two-year period [17]. On the other hand, two studies have reported an association. Jonsdottir et al. found that both IgM and IgG ACL antibodies occurred more frequently in patients who received TNF *α* inhibitors for three months or longer and that positivity was associated with worse clinical outcomes [21]. Finally, Petitpain et al. reported 85 spontaneous cases of TNF *α* inhibitor-related thromboembolic events from 2000 to 2006 [22]. These events occurred in patients with few or no traditional risk factors for VTE [22]. Unfortunately, the study is limited by variations in how some data, like antibody testing, were assessed and reported. Of the 85 patients, only 23 had documented antibodies, and of those only four had APLs [22].

We believe our findings strengthen the evidence supporting an association of TNF *α* inhibition with APL antibody induction and VTE. Our series, similar to Petitpain's, identified venous thrombosis in patients with few or no risk factors other than APL. Three of the patients had received etanercept for three years, and a fourth had been on the medication previously. These patients would not have been identified by Ferraro-Peyret, whose analysis was limited to a two-year window of therapy. Strikingly, all five of our cases occurred over a five-year period, in a single private clinic of approximately 500 patients. Unfortunately, four of the five patients did not receive baseline APL testing.

What might be the implications of an association of anti-TNF *α* therapy with APL antibody induction and VTE? Others have pondered whether patients should be screened for APLs prior to initiating TNF *α* inhibitor therapy [1, 12, 22, 23, 25], but this is rarely done in the absence of other risk factors for APS [22]. Ramos-Casals et al. recommend performing baseline immunologic studies including ANA, anti dsDNA, APLs, ANCA, and chest X-ray based upon their retrospective study of 233 cases of autoimmune diseases associated with TNF *α* inhibitor therapy. They also suggest tailoring therapy based upon the severity of autoimmune features. For example, for patients with preexisting autoimmune diseases, Ramos-Casals et al. recommend using anti-TNF *α* agents with caution.

## 4. Conclusion

Our case series supports the association between etanercept, APLs, and VTE. We believe that testing for APLs prior to initiation of anti-TNF therapy is reasonable, given the evidence described herein and the morbidity and mortality attendant to VTE. A cohort study that included baseline autoantibody screening could define the benefits and cost of this strategy. As the number of new classes of biologic DMARDS increases, it is also imperative for us to obtain a better understanding of the mechanism of induction of autoantibodies and autoimmunity with these agents.
